# Long-term continuous instrumented insole-based gait analyses in daily life have advantages over longitudinal gait analyses in the lab to monitor healing of tibial fractures

**DOI:** 10.3389/fbioe.2024.1355254

**Published:** 2024-03-01

**Authors:** Elke Warmerdam, Christian Wolff, Marcel Orth, Tim Pohlemann, Bergita Ganse

**Affiliations:** ^1^ Werner Siemens-Endowed Chair for Innovative Implant Development (Fracture Healing), Departments and Institutes of Surgery, Saarland University, Homburg, Germany; ^2^ German Research Center for Artificial Intelligence (DFKI), Saarbrücken, Germany; ^3^ Department of Trauma, Hand and Reconstructive Surgery, Departments and Institutes of Surgery, Saarland University, Homburg, Germany

**Keywords:** digital medicine, fracture, ground reaction force, injury, pedography, postoperative treatment, rehabilitation, wearable sensors

## Abstract

**Introduction:** Monitoring changes in gait during rehabilitation allows early detection of complications. Laboratory-based gait analyses proved valuable for longitudinal monitoring of lower leg fracture healing. However, continuous gait data recorded in the daily life may be superior due to a higher temporal resolution and differences in behavior. In this study, ground reaction force-based gait data of instrumented insoles from longitudinal intermittent laboratory assessments were compared to monitoring in daily life.

**Methods:** Straight walking data of patients were collected during clinical visits and in between those visits the instrumented insoles recorded all stepping activities of the patients during daily life.

**Results:** Out of 16 patients, due to technical and compliance issues, only six delivered sufficient datasets of about 12 weeks. Stance duration was longer (*p* = 0.004) and gait was more asymmetric during daily life (asymmetry of maximal force *p* < 0.001, loading slope *p* = 0.001, unloading slope *p* < 0.001, stance duration *p* < 0.001).

**Discussion:** The differences between the laboratory assessments and the daily-life monitoring could be caused by a different and more diverse behavior during daily life. The daily life gait parameters significantly improved over time with union. One of the patients developed an infected non-union and showed worsening of force-related gait parameters, which was earlier detectable in the continuous daily life gait data compared to the lab data. Therefore, continuous gait monitoring in the daily life has potential to detect healing problems early on. Continuous monitoring with instrumented insoles has advantages once technical and compliance problems are solved.

## 1 Introduction

Bone healing is a process that requires appropriate biomechanical conditions (Barcik and Epari, 2021) and blood supply (Lu et al., 2007; Keramaris et al., 2008), and involves numerous cells and molecular mechanisms (Saul et al., 2023). Long-bone fractures fail to heal in up to 5%–10% of cases (Zura et al., 2016). Methods to monitor progress in fracture healing that are routinely used in clinical practice today include the clinical impression, questionnaires, and infrequent radiographic imaging (Mundi et al., 2020). However, none of these methods reliably allows early prediction of non-unions or delays in healing, and the timely start of an intervention (Blokhuis et al., 2001; Ganse et al., 2022). Possible interventions include the application of low-intensity pulsed ultrasound (Leung et al., 2004), extracorporeal shock-wave therapy (Searle et al., 2023), magnetic fields (Ribeiro et al., 2023), or revision surgery (Giannoudis et al., 2015). When healing delays occur, rehabilitation times are often extensive, immobilization can last several months, and there are massive negative socio-economic and psychological effects (Simpson and Tsang, 2017).

In the last few years, the number of studies that used gait analysis to monitor fracture healing has increased (Bennett et al., 2021; Larsen et al., 2021; Kröger et al., 2022). For longitudinal monitoring of the healing progress, spatiotemporal gait parameters, kinematics, kinetics, and pedography-parameters were suitable (Warmerdam et al., 2023). Gait analysis can allow for better fracture healing monitoring, as well as timely individualized rehabilitation and treatment. Particularly, changes in gait speed and asymmetry measures have great potential to indicate problems in fracture healing in a more objective and timelier manner (Warmerdam et al., 2023). However, these supervised lab assessments take place in an artificial setting where patients are aware that they are being observed, and therefore patients may not show their usual gait pattern (Brodie et al., 2016; Warmerdam et al., 2020). It is known that gait parameters, such as gait speed and step length are lower when measured during daily life, whereas temporal gait parameters are higher compared to lab assessments (Warmerdam et al., 2020).

In the last decade, many instrumented insoles were developed to monitor gait (Ngueleu et al., 2019; Wang et al., 2019; Ma et al., 2023). With recent technological advances, it has become possible to collect gait data continuously during daily life. It is now possible to conduct measurements in a more natural context throughout the rehabilitation period (Braun et al., 2017). Improvements in battery life and data storage capacity, durability, usability, hysteresis and drift, and higher sample frequencies made long-term measurements with a high time resolution possible (Elstub et al., 2022; Subramaniam et al., 2022; Wolff et al., 2023). A recording frequency of 25 Hz and more allows for analyses of the vertical ground reaction force curve with its characteristic two maxima and in-between minimum during the stance-phase (Wolff et al., 2023). With lower recording frequencies, such as of the insoles typically used to continuously monitor patients with diabetes in their daily lives, such analyses were previously not possible. With lower recording frequencies, peak force spikes were missed (Abbott et al., 2019).

We conducted a longitudinal prospective observational clinical trial to compare insole-based lab assessments with continuous recordings from the daily life in patients throughout the healing phase after tibial fractures. Our hypotheses were i) That differences exist between measurements in the lab compared to continuous recordings during the daily life of patients, that ii) The daily life gait data improve over time, and that iii) Long-term continuous measurements can deliver high-resolution data to monitor the healing process in more detail.

## 2 Material and methods

The prospective cohort study is part of the project Smart Implants 2.0—Weight-bearing and Gait Observation for Early Monitoring of Fracture Healing and Individualized Therapy after Trauma, funded by the Werner Siemens Foundation. It was registered in the German Clinical Trials Register (DRKS-ID: DRKS00025108). Ethical approval was obtained from the IRB of Saarland Medical Board (Ärztekammer des Saarlandes, Germany, application number 30/21). The study was conducted with oral and written informed consent according to the newest version of the Declaration of Helsinki. In this observational study, measurements in the lab were only conducted during inpatient stays and when outpatient clinic appointments were scheduled anyway to not alter the frequency of consultations and degree of care. All patients enrolled in this study received both types of measurements simultaneously, longitudinal intermittent measurements in the lab with every inpatient or outpatient stay in the hospital, and continuous daily life measurements throughout the first 3 months after fracture.

### 2.1 Inclusion and exclusion criteria

Recruitment took place in a monocenter study at Saarland University Hospital in Germany between February 2022 and June 2023. Inclusion criteria were patients of 18 years or more of both sexes with tibial fractures. Exclusion criteria were age under 18 years, immobility already before the fracture event, use of walking aid prior to the fracture, inability to give consent, further major injuries of the lower extremities in addition to the tibial fracture, alcohol or drug abuse, pregnancy, and participation in another ongoing clinical study within the past month or less.

### 2.2 Measurements in the lab

**FIGURE 1:**
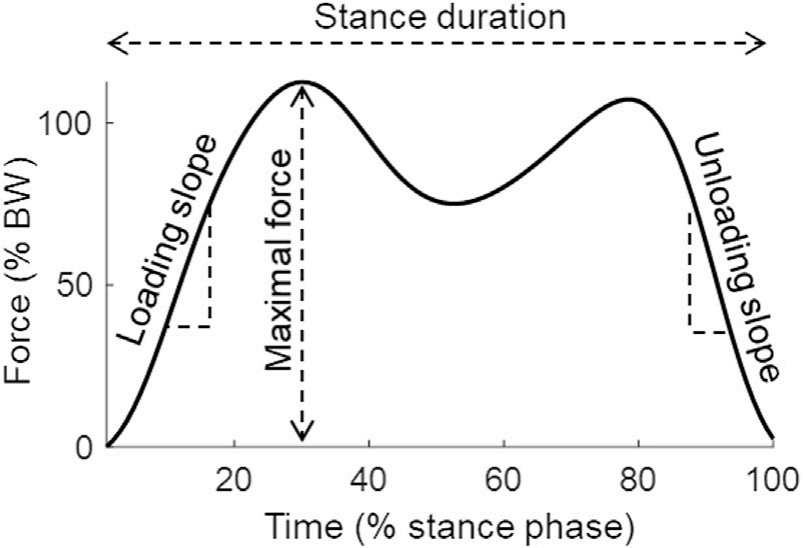
Vertical ground reaction force curve of a stance phase during walking.

Instrumented pedography insoles (OpenGO, Moticon GmbH, Munich, Germany) equipped with 16 pressure sensors were matched for shoe size of the patient and calibrated to the patient’s body weight. Data were recorded with 100 Hz from both feet with one insole in each shoe. The insole sensors have a resolution of 0.25N/cm^2^ and are known to underestimate the extrema in the force data by −3.7% to −1.1% at 100 Hz (Cramer et al., 2022). All patients were asked to walk straight for 10 m. Ten steps in the middle part of the walk were extracted for data analysis. Only the vertical ground reaction force data were taken into account. The force values are around zero at the beginning and the end of the stance phase. There are generally two maxima with one minimum in between during walking. The first maximum represents the loading force, the minimum represents the mid-stance phase and the second maximum represents the push-off force (Figure 1).

### 2.3 Measurements during the daily life

During the day life, the same insoles were used for the measurements, but data were recorded at a rate of 25 Hz. Due to limited data storage capacity, 25 Hz is the maximum recording frequency these insoles provide for continuous measurements. The data are primarily stored on the insoles, and patients need to actively conduct data transfer to a smartphone using an app once daily. In addition, the insole batteries need to be changed and charged on a daily basis. Patients were trained to use the insoles, store the data, and charge and change the batteries. The insoles were activated the entire time all day and night, and they recorded every activity when worn. Only stepping activities were extracted from the data for analysis, however no distinctions were made between different types of walking, e.g., indoor, outdoor, stairs.

### 2.4 Data processing

As the initial processing step, stance phases of the gait cycles were identified and extracted from the time-series data. Stance phases were determined by considering any activity with consecutive ground reaction force readings above 30 N. To account for possible recording-device faults, a tolerance of up to three missing values was allowed. Additionally, load-bearing activities lasting less than 300 ms or more than 3500 ms were discarded. These thresholds were arbitrarily chosen after exploring the data.

To ensure comparability between subjects and steps, the force was normalized to the body weight of the patients and the time to the duration of the stance phase using equidistant subsampling on a Cubic Spline interpolation. Because of the low recording frequency and the sensor noise, a Gaussian filter (Sigma = 3, kernel size 7) was applied to the raw data. Where using the filtered data still resulted in inconclusive extremum candidates, we implemented additional detection strategies in the following order: First, extremum candidates occurring within the first or last 10% of the stance phase were eliminated. Then, events in the second half of the stance phase were eliminated as candidates for the first maximum and *vice versa* for the second. This check split the maxima candidates into two groups for first and second maximum candidates, respectively. Next, if multiple extremum candidates occurred within a pool size of 5% of the stance phase or either group contained one candidate with a force reading greater than all other candidates within the group by a factor of 1.05, this candidate with the highest force value was selected. If this procedure delivered too few candidates, previous eliminations were reinstated based on their highest achieved monotony distance, until the desired number of candidates was reached.

Any stance activity that after the application of these strategies during step detection had an irregular number of unambiguous extremum candidates was classified as a non-step event and subsequently removed from the dataset. From all detected steps, the maximal force and stance duration were extracted from the raw data. The loading slope and unloading slope were only extracted when there were two maxima during the stance phase. Daily averages were calculated for these parameters. For the analysis of the average gait parameters, only the values of the injured side were used. The asymmetry of the four parameters was calculated with data of the injured and the healthy side according to the following equation:

$$Asymmetry=\frac{healthy\;side-injured\;side}{\left(healthy\;side+injured\;side\right)/2}*100$$

An asymmetry value of 0% indicates perfect symmetry. Additionally, the number of steps per day and the average walking bout length were extracted, where both sides were taken into account (Table 1).

**TABLE 1:** Description of the parameters analyzed in this study.

Parameter	Description	Unit
Maximal force	Force maximum during the stance phase	%Body weight
Stance duration	Consecutive force reading above 30 N	Seconds
Loading Slope	Slope of the line drawn between the start of the load activity and the first force value equal or higher than 80% of the first extremum	%Body weight/%Time
Unloading Slope	Slope of the line drawn between the first force value equal or lower than 80% of the second extremum and the end of the load activity	%Body weight/%Time
Number of steps	Total number of steps measured per day	Steps
Length of walking bout	Number of steps per walking bout. Steps belonged to one walking bout if consecutive steps were within 5 s from each other	Steps

### 2.5 Statistical analyses

To compare the lab assessments with the continuous daily life data, an average of 7 days of the continuous data was calculated around the time of the lab assessment. All lab data were compared with the matching continuous seven-days-average data. The data were tested for normality with the Shapiro-Wilk test. In case of a normal distribution, a Student’s t-test was used, otherwise a Wilcoxon signed-rank test was used to compare the lab data with continuous data.

To analyze whether the continuous data improved throughout the healing process, the data of the first week, the sixth week and the last week of patients with union were compared with a repeated measures ANOVA with time as a within-patient factor. Post-hoc tests were performed with Bonferroni correction. Significance was assumed at *p* < 0.05 for all tests.

## 3 Results

### 3.1 Enrolment

**FIGURE 2:**
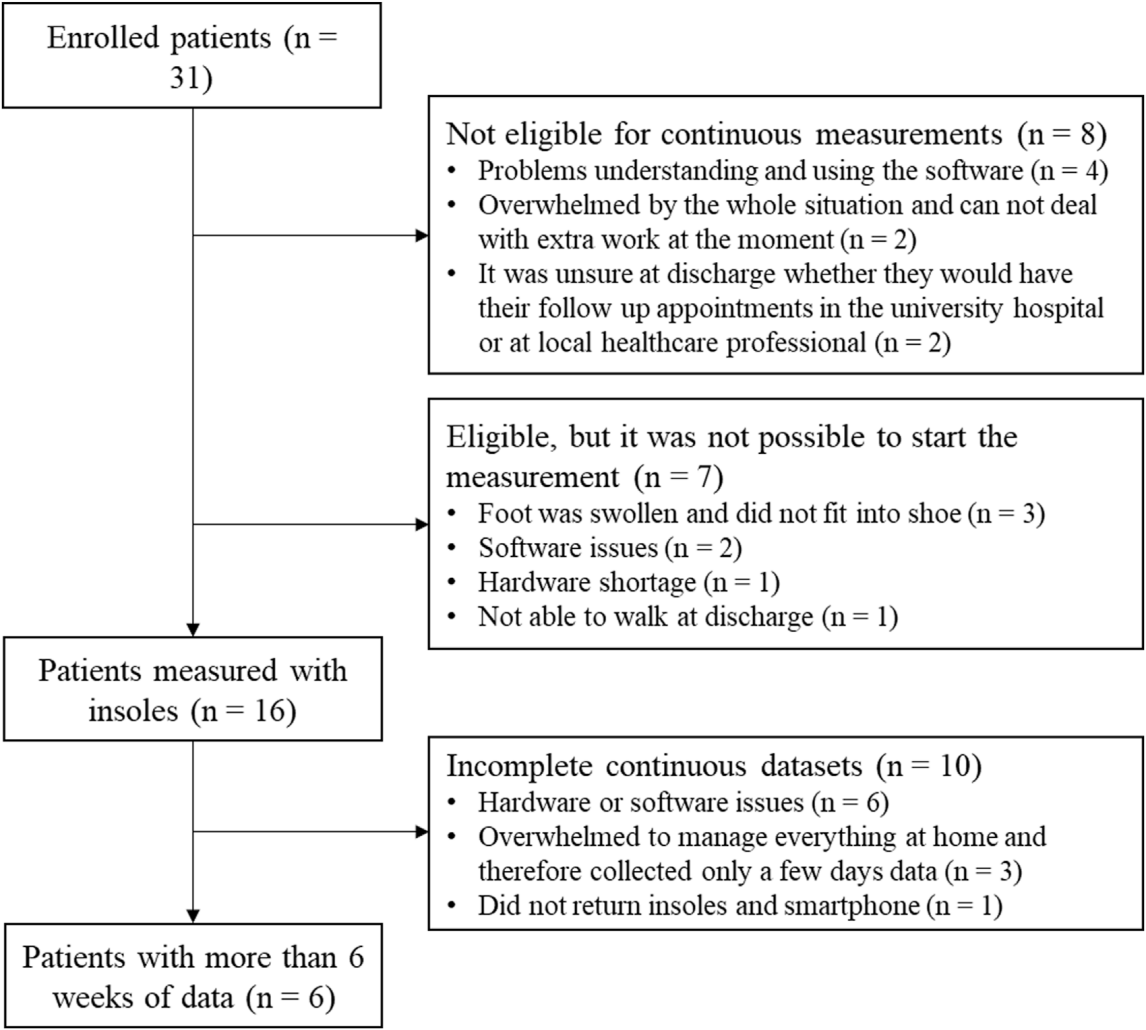
One Flow chart of patients enrolled in the study with information about the exclusion for the continuous data analysis.

Thirty-one patients with a tibial shaft or proximal tibial fracture were enrolled in this study between February 2022 and May 2023. Eight patients were not eligible to participate in the continuous measurements and seven patients only took part in the lab assessments, as they were unable to start the continuous measurements for either technical or compliance reasons. Out of the sixteen patients who collected continuous data, only six data sets contained enough data (>6 weeks) to analyze. More detailed information can be found in Figure 2.

### 3.2 Intermittent lab vs. continuous daily-life data

**FIGURE 3:**
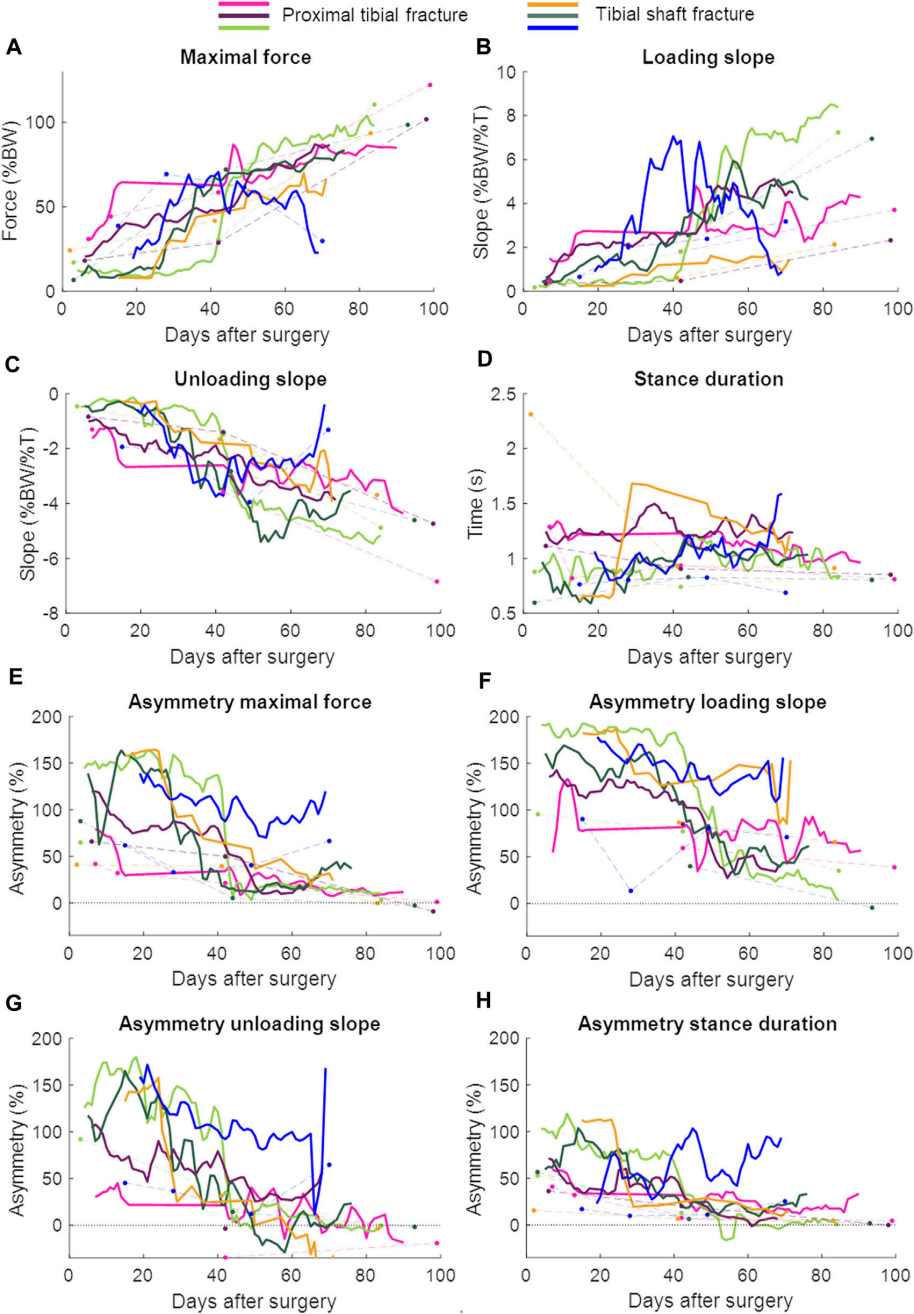
Continuous daily life (solid lines) and lab data (dots connected by dashed lines) with each patient in a different color. The continuous daily-life data are presented as a 3-day moving average. The patient represented with the blue color developed an infected non-union. BW = body weight; T = time. **(A)** Maximal force, **(B)** Loading slope, **(C)** Unloading slope, **(D)** Stance duration, **(E)** Asymmetry of the maximal force, **(F)** Asymmetry of the loading slope, **(G)** Asymmetry of the unloading slope, **(H)** Asymmetry of the stance duration.

**FIGURE 4:**
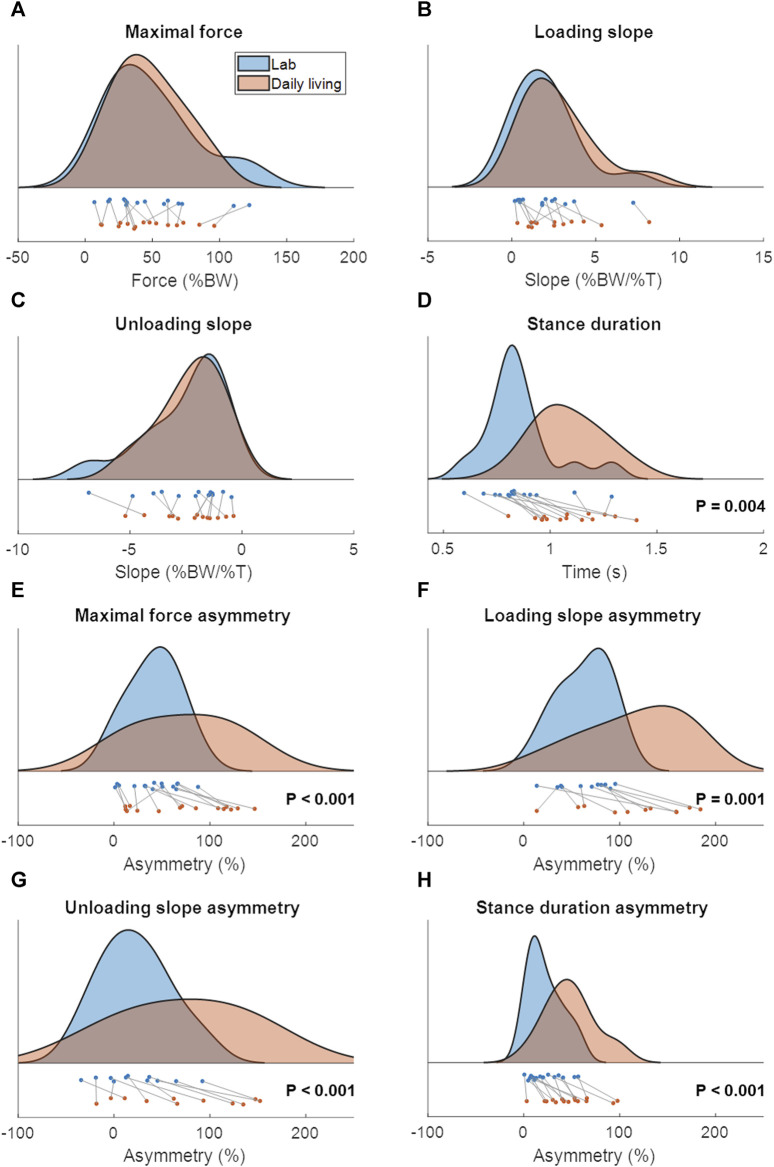
The distribution and boxplot of the lab data (blue) and continuous daily-life data (coral). *p*-values are written in each subfigure in case of a significant difference. The data in c and d were not normally distributed. **(A)** Maximal force, **(B)** Loading slope, **(C)** Unloading slope, **(D)** Stance duration, **(E)** Asymmetry of the maximal force, **(F)** Asymmetry of the loading slope, **(G)** Asymmetry of the unloading slope, **(H)** Asymmetry of the stance duration.

Patient characteristics are shown in Table 2 and the lab and continuous gait data are represented in Figure 3. The lab data were compared to the average of 1 week of continuous data around the same days of the lab assessment. The stance duration and all asymmetry parameters were significantly different between walking in the lab and everyday walking (Figure 4).

**TABLE 2:** Patient characteristics.

Parameter	Value
N (%female)	6 (33%)
Age (years, mean ± standard deviation)	52 ± 14
Height (m, mean ± standard deviation)	1.78 ± 0.15
Weight (kg, mean ± standard deviation)	82 ± 17
Fracture type (proximal tibia/tibial shaft)	3/3
Fracture side (left/right)	4/2

### 3.3 Changes in gait throughout the healing process of tibial fractures

**FIGURE 5:**
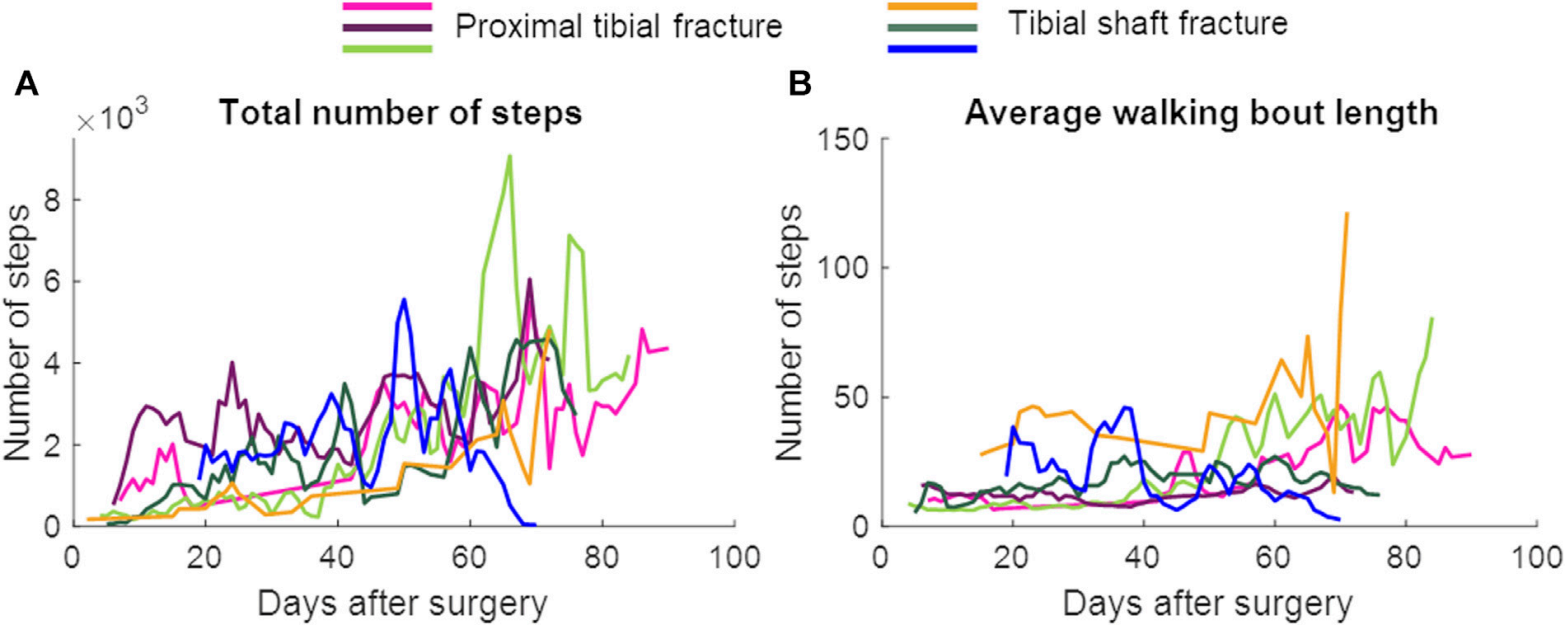
The total number of steps and the average walking bout length per day. Data are presented as a 3-day moving average. The patient represented with the blue color developed an infected non-union. **(A)** Total number of steps, **(B)** Average walking bout length.

In patients with union, a repeated measures ANOVA showed that the continuous daily-life data improved significantly during the first 3 months of the healing process, except for the stance duration (Table 3; Figure 3; Figure 5). Post-hoc testing revealed that all parameters that significantly improved showed this improvement between the first and last recorded week. The maximal force, unloading slope and the number of steps per day showed significant improvements during the first and second 6-week periods. Asymmetry of the maximal force decreased significantly during the first 6 weeks only (Table 3).

**TABLE 3:** *p*-values of repeated measures ANOVA to evaluate changes in parameters from daily life data between the first week, sixth week and last week of data collection of the five patients with union. Post-Hoc tests were performed with Bonferroni correction. Significant *p*-values are shown in bold.

	*p*-value repeated measures ANOVA	*p*-value first week vs. week six	*p*-value first week vs. last week	*p*-value week six vs. last week
Maximal force	**<0.001**	**0.006**	**<0.001**	**0.007**
Loading slope	**0.008**	0.394	**0.008**	0.098
Unloading slope	**<0.001**	**0.006**	**<0.001**	**0.005**
Stance duration	0.167			
Asymmetry maximal force	**0.004**	**0.011**	**0.007**	1.000
Asymmetry loading slope	**0.011**	0.433	**0.012**	0.085
Asymmetry unloading slope	**0.007**	0.068	**0.008**	0.315
Asymmetry stance duration	**0.020**	0.106	**.024**	0.827
Number of steps per day	**<0.001**	**0.003**	**<0.001**	**<0.001**
Number of steps per walking bout	**0.043**	0.941	**0.048**	0.254

## 4 Discussion

The present study showed that the stance duration and the asymmetry parameters were significantly different between lab assessments and continuous daily life monitoring. Only six out of 31 attempts to collect sufficient continuous gait data in the daily life of patients were successful, while the others failed either due to hard- or software, or compliance issues. The data obtained were of high quality and allowed earlier detection of union problems. Thus, continuous monitoring has advantages once technical and compliance problems are solved.

Differences between laboratory-based and continuous daily life measurements were expected based on previous studies that compared similar gait parameters between lab and daily life assessments in healthy (older) adults and several other, mainly neurological, patient groups (Toosizadeh et al., 2015; Del Din et al., 2016; Storm et al., 2018; Hillel et al., 2019; Takayanagi et al., 2019; Van Ancum et al., 2019; Rast et al., 2022). However, in our study, force-related parameters were not significantly different between the lab and the daily life. It appears that during daily life, when patients walk on crutches or still have pain, they focus more on their walking. In patients with hip osteoarthritis during two, three and four-point gait with crutches, forces in the hip joint were 13, 17% and 12% reduced, respectively (Damm et al., 2013). Additionally, gait speed in patients with Parkinson’s disease was lower when walking with a cane compared to walking without walking aids (Bryant et al., 2012). The more focused and cautious gait when using crutches may lead to a higher comparability of everyday life to lab-assessment data. This seems to be the case especially in the first weeks after injury and/or surgery.

The asymmetry was significantly different between lab assessments and continuous monitoring in daily life. In patients with Parkinson’s disease, a significant difference in step length asymmetry was found between lab and daily-life assessments, however, there were no differences in asymmetry of temporal parameters (Del Din et al., 2016). The differences between lab and everyday life assessments could at least partially be due to the variance in environment. In the lab, patients walk in a straight line, where during the daily life patients more often walk along a curved path or turn on the spot, especially when they walk indoors. During outdoor walking, patients might come across different types of surfaces, which might not be as smooth, solid and level as in the lab. This is known to alter the gait pattern and its variability (Thies et al., 2005; Nohelova et al., 2021), but could potentially also have an effect on asymmetry. The disadvantage of daily life assessments is that it is unknown what exactly the patients have done, as movements are not standardized. The step-detection algorithm should only extract steps, but just from the insole data, it cannot be concluded whether someone is stepping around in the kitchen during cooking, is walking outside or is walking on the stairs. It is expected that most detected steps are from ‘regular’ walking (covering a horizontal distance), there will be a certain number of steps that are measured during other activities. Furthermore, non-step load bearing events might falsely present as steps. The fact that all extracted steps were included in the data analysis has probably led to more variability compared to the lab assessment. On the other hand, a greater number of steps was collected and thereby more detailed and realistic information obtained. Depending on what the patients did and how they felt, there may be greater fluctuations, but trends can be identified by averaging the values.

In the present study, gait during the daily life significantly improved throughout the first 3 months after tibial fractures. To the best of our knowledge, only two studies have used pressure-sensing insoles to monitor patients after an injury during their daily life. A feasibility study in patients with ankle fractures showed an increase in force and activity (Braun et al., 2016). In the other study, step count and time spent walking had a larger impact than the maximal force and cadence on patient-reported physical function 1 year after a lower leg fracture (North et al., 2023). Both studies only measured up until 6 weeks after surgery. Since proximal tibial fractures need about 3 months to heal (LI et al., 2023), in the present study patients were monitored for 3 months after surgery.

The larger data quantity from the continuous daily-life data could make it easier to detect healing problems early. One out of the six patients developed a non-union caused by bacterial infection involving the soft tissues and fracture. The last measurement was performed when the patient was readmitted to the hospital because of the infection (70 days after surgery, Figure 3). Especially in the loading and unloading slope of the force curve, a decrease in performance could be seen earlier in the daily-life data compared to the lab data (Figure 3). If the data were stored on a cloud and medical professionals had access to them, they could receive an automatic notification if the performance decreased, as the pattern of change appears to be distinct enough to be detectable by either traditional reasoning approaches or machine learning. The medical professional could closely monitor the data remotely and invite the patient to the clinic earlier to adjust treatment, if necessary.

The insoles require a lot of handling from the patients. Every day, the patients are required to save the data and change and recharge the batteries. Many patients were already overwhelmed by the new situation after their injury and could not handle the extra work they had to put in the insoles. In addition, we experienced a high rate of hardware failure, which led to the approximate consumption of insole hardware with a value of 2,000 to 4,000 Euros per patient in the present study. Out of 23 patients who were eligible to participate in the continuous measurements in their everyday lives, despite our best efforts, only 6 patients collected a data set that was of value to us. Another limitation of measuring with the insoles is that the number of steps per day measured is likely not the total number of steps taken per day. This is the case because the patients might not have worn the insoles all day. Especially at home, they might not walk around with their shoes on. In addition, as the insoles inactivate during breaks and will re-activate only after a few steps, these first few steps will not be recorded. These factors may have affected the average walking bout length and might have had a minor influence on the other gait parameters.

These difficulties in data collection by instrumented insoles may either be overcome when better and more suitable hardware becomes available, or by collecting data via the implant used to treat the fracture itself (Ganse et al., 2022; Windolf et al., 2022). Automated data collection of fracture stiffness via implants may deliver a similar force curve and allow for direct measurements less reliant on patient compliance. In the future, such systems may allow for much more accurate healing prediction and individualized rehabilitation monitoring than the present insole-based approach. Immediate patient feedback systems, such as via alarms (sound, vibration) when weight-bearing restrictions are exceeded may complement the functionalities (Ganse et al., 2016; Abbott et al., 2019).

## 5 Conclusion

Differences exist between laboratory and continuous assessments, mainly in the gait asymmetry parameters caused by a different and more diverse behavior at home. There were no differences in force-related parameters between laboratory and home measurements, potentially due to more careful gait with crutches, as well as pain. The continuous assessments in the daily life provided gait data with a higher time resolution, which makes it easier to detect changes in performance sooner. Therefore, continuous gait monitoring in the daily life has potential to detect healing problems early on. Future research should focus on finding a more feasible device to monitor patients during daily life, and on the identification of gait parameters that serve to differentiate early between patients with union and patients with healing problems.

## Data Availability

The raw data supporting the conclusion of this article will be made available by the authors, without undue reservation.
